# Neurocognitive and psychiatric disorders‐related axonal degeneration in Parkinson's disease

**DOI:** 10.1002/jnr.24584

**Published:** 2020-02-05

**Authors:** Christina Andica, Koji Kamagata, Taku Hatano, Yuya Saito, Wataru Uchida, Takashi Ogawa, Haruka Takeshige‐Amano, Akifumi Hagiwara, Syo Murata, Genko Oyama, Yashushi Shimo, Atsushi Umemura, Toshiaki Akashi, Akihiko Wada, Kanako K. Kumamaru, Masaaki Hori, Nobutaka Hattori, Shigeki Aoki

**Affiliations:** ^1^ Department of Radiology Juntendo University Graduate School of Medicine Tokyo Japan; ^2^ Department of Neurology Juntendo University Graduate School of Medicine Tokyo Japan; ^3^ Department of Radiological Sciences Graduate School of Human Health Sciences Tokyo Metropolitan University Tokyo Japan; ^4^ Department of Neurosurgery Juntendo University Graduate School of Medicine Tokyo Japan; ^5^ Department of Radiology Toho University Omori Medical Center Tokyo Japan

**Keywords:** axons, biomarkers, diffusion tensor imaging, linked independent component analysis, neurite orientation dispersion and density imaging, Parkinson's disease

## Abstract

Neurocognitive and psychiatric disorders have significant consequences for quality of life in patients with Parkinson's disease (PD). In the current study, we evaluated microstructural white matter (WM) alterations associated with neurocognitive and psychiatric disorders in PD using neurite orientation dispersion and density imaging (NODDI) and linked independent component analysis (LICA). The indices of NODDI were compared between 20 and 19 patients with PD with and without neurocognitive and psychiatric disorders, respectively, and 25 healthy controls using tract‐based spatial statistics and tract‐of‐interest analyses. LICA was applied to model inter‐subject variability across measures. A widespread reduction in axonal density (indexed by intracellular volume fraction [ICVF]) was demonstrated in PD patients with and without neurocognitive and psychiatric disorders, as compared with healthy controls. Compared with patients without neurocognitive and psychiatric disorders, patients with neurocognitive and psychiatric disorders exhibited more extensive (posterior predominant) decreases in axonal density. Using LICA, ICVF demonstrated the highest contribution (59% weight) to the main effects of diagnosis that reflected widespread decreases in axonal density. These findings suggest that axonal loss is a major factor underlying WM pathology related to neurocognitive and psychiatric disorders in PD, whereas patients with neurocognitive and psychiatric disorders had broader axonal pathology, as compared with those without. LICA suggested that the ICVF can be used as a useful biomarker of microstructural changes in the WM related to neurocognitive and psychiatric disorders in PD.

## Graphical abstract

This study demonstrates that axonal loss, indicated by a reduction in the intracellular volume fraction, is a major factor underlying white matter pathology related to neurocognitive and psychiatric disorders in Parkinson's disease. Furthermore, patients with neurocognitive and psychiatric disorders had broader axonal pathology (posterior predominant) compared with those without such disorders.

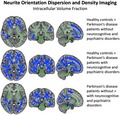

**Significance**

Using neurite orientation dispersion and density imaging, this study demonstrates that axonal loss, indicated by a reduction in the intracellular volume fraction (ICVF), is a major factor underlying white matter (WM) pathology related to neurocognitive and psychiatric disorders in Parkinson's disease (PD). However, patients with neurocognitive and psychiatric disorders had broader axonal pathology (posterior predominant) compared with those without such disorders. Linked independent component analysis suggested that ICVF had the highest contribution to the diagnosis, thus representing a useful biomarker to detect microstructural changes in the WM related to neurocognitive and psychiatric disorders in PD.

## 1 INTRODUCTION

Parkinson's disease (PD) is primarily considered to be a neurodegenerative movement disorder that is characterized by bradykinesia, rigidity, resting tremor, and postural abnormalities, with the pathological hallmarks of the loss of dopaminergic neurons in the substantia nigra (Fearnley & Lees, 1991) and the widespread aggregation of α‐synuclein in the form of Lewy pathology (Braak et al., 2003). In addition to dopamine‐related impairments, it has become evident that PD involves multiple neurotransmitter pathways, including the noradrenergic, serotonergic, and cholinergic systems, within the brain that are associated with a wide variety of motor and non‐motor symptoms (Schapira, Chaudhuri, & Jenner, 2017). Among the non‐motor symptoms, neurocognitive and psychiatric symptoms (NCPs) have received attention because these signs are highly prevalent, contribute to severe disability (Weintraub, Moberg, Duda, Katz, & Stern, 2004), impair quality of life (Barone et al., 2009), and shorten life expectancy (Chaudhuri, Healy, Schapira, & National Institute for Clinical Excellence, 2006).

Defects to the structure of the white matter (WM) are associated with many disorders affecting cognition and physiological states (Fields, 2008). Diffusion tensor imaging (DTI) has been widely used to investigate pathological changes in the WM of PD patients by probing the diffusivity of water molecules within the WM tracts (Cochrane & Ebmeier, 2013). Previous studies that utilized DTI to evaluate the WM of PD patients with NCPs reported conflicting observations, such as lower fractional anisotropy (FA) and higher mean diffusivity (MD) (Agosta et al., 2014; Hattori et al., 2012; Huang et al., 2014; Yao et al., 2016; Zhong et al., 2013), as well as unaltered FA and MD (Melzer et al., 2013; Surdhar et al., 2012). Sample size, demographic, and clinical data variances, however, are likely to have contributed to these discrepant findings but may also be due to the limitations of DTI. First, DTI assumes that water diffusion has a Gaussian probability distribution (Basser & Jones, 2002). However, water molecules in biological structures are hindered by barriers, such as the cell membrane and the internal membranes that encase the organelles; thus, DTI is not appropriate for modeling of non‐Gaussian diffusion in living tissues (Assaf & Pasternak, 2008). Second, DTI‐derived indices have been shown to be sensitive, but not specific, to microstructural changes. A decrease in FA accompanied by an increase in MD may be attributed to reduced axon density, increased axonal dispersion, and demyelination (Assaf & Pasternak, 2008).

On the contrary, neurite orientation dispersion and density imaging (NODDI) can be used to address the limitations of DTI. NODDI was developed on the basis of a multicompartment (intracellular, extracellular, and cerebrospinal fluid) diffusion model with the particular advantage of providing useful microstructural indices from multishell diffusion magnetic resonance imaging (MRI) data that can be acquired in a clinically feasible amount of time (Zhang, Schneider, Wheeler‐Kingshott, & Alexander, 2012). The intracellular volume fraction (ICVF) and the orientation dispersion index (ODI) are indices of NODDI that are assumed to reflect the packing density of axons in WM and the spatial organization of the axons, respectively, which are two disentangled facets of FA (Zhang et al., 2012). Previous studies have demonstrated the superiority of NODDI over DTI for the detection of PD pathology in the substantia nigra (Kamagata et al., 2016), nigrostriatal pathway (Andica et al., 2018), and gray matter (GM) (Kamagata et al., 2017). A simplified model of NODDI, neurite density imaging, has also been used to demonstrate reduction in the density of some WM areas of patients with PD (Surova et al., 2016).

In this study, we used NODDI to characterize in vivo WM pathology associated with the NCPs of PD patients. Using tract‐based spatial statistics (TBSS) and tract‐of‐interest (TOI) analyses we evaluated differences in NODDI metrics between healthy controls and PD patients with and without NCPs in addition to DTI metrics. Also, recent studies of PD patients (Aquino et al., 2014; Kamagata et al., 2017; Melzer et al., 2015) have reported multimodal MRI data in terms of a robust biomarker to further elucidate the pathology of the disease. Those data, however, were analyzed separately and only detected single‐dimensional information by each measure; thus, as a consequence, failed to model common variance across features. In an attempt to overcome these limitations, linked independent component analysis (LICA) was applied in the present study (Groves, Beckmann, Smith, & Woolrich, 2011; Groves et al., 2012) as an integrated approach by fusing different modalities to model inter‐subject variabilities across the measured indices.

## 2 METHODS

### 2.1 Study participants

The cohort of this retrospective case–control study included 39 PD patients who remained free from atypical parkinsonism and exhibited a good response to anti‐parkinsonian therapy for 18 months or more after diagnosis. PD was diagnosed by three movement disorders specialists (TH, GO, and YS) based on the clinical diagnostic criteria for PD of the Movement Disorder Society (MDS; Postuma et al., 2015). At the time of MRI and clinical examination, all PD patients were taking levodopa in combination with a dopamine decarboxylase inhibitor (benserazide or carbidopa). Disease severity was then assessed on the basis of the non‐motor and motor scores of the MDS‐Unified Idiopathic PD Rating Scale (MDS‐UPDRS) parts I and III, respectively (Goetz et al., 2008).

The NCPs of PD patients might be caused by overlapping pathophysiology, whereas some symptoms might be similar (Fields, 2017). Thus, PD patients were categorized into groups with or without NCPs (PD‐wNCPs and PD‐woNCPs, respectively), where the total MDS‐UPDRS score of items I.1–I.6 (I.1, Cognitive impairment; I.2, Hallucinations; I.3, Depression; I.4, Anxiety; I.5, Apathy; and I.6, Dopamine dysregulation syndrome) was equal to ≥1 (PD‐wNCPs; *n* = 20; 12 males; mean age, 70.15 ± 4.03 years) or 0 (PD‐woNCPs; *n* = 19; 6 males; mean age, 67.21 ± 8.16 years), respectively. Twenty‐five age‐ and sex‐matched healthy controls (10 males; mean age, 67.88 ± 2.11 years) with no history of neurologic or psychiatric diseases and no abnormal signals on structural MRI were recruited as a control group. The demographic and clinical characteristics of all participants are shown in Table 1. The study protocol was approved by the Institutional Review Board of Juntendo University Hospital, Tokyo, Japan, and written informed consent was obtained from each participant.

**Table 1:** Demographic characteristics of the participants

	HC	PD‐woNCPs	PD‐wNCPs	*p* value
Number	25	19	20	N/A
Age, mean ± *SD* (years)^a^	67.88 ± 2.11	67.21 ± 8.16	70.15 ± 4.03	0.18
Sex (male/female)^b^	10/15	6/13	12/8	0.18
Disease duration, mean ± *SD* (years)^c^	N/A	9.84 ± 6.21	9.95 ± 7.69	0.79
Hoehn and Yahr scale (1/2/3/4/5), number^b^	N/A	3/11/4/1/0	2/9/7/2/0	0.68
MDS‐UPDRS part I.1‐I.6, mean ± *SD^c^*	N/A	0	2.90 ± 2.49	0.000000012
MDS‐UPDRS part III, mean ± *SD* ^c^	N/A	17.42 ± 12.28	18.60 ± 9.99	0.55
Total LED, mean ± *SD* ^d^	N/A	908.84 ± 444.54	817.85 ± 469.99	0.54
Total white matter volume, mean ± *SD* (ml)^a^	449.02 ± 53.98	452.92 ± 44.88	468.58 ± 50.11	0.57

Note

Statistical analyses were performed using ^a^one‐way ANOVA test, ^b^chi‐squared test, ^c^Mann–Whitney test, or ^d^an unpaired *t*‐test.

Abbreviations: HCs, healthy controls; LED, levodopa equivalent dose; MDS‐UPDRS, the Movement Disorder Society‐Unified Parkinson's Disease Rating Scale; N/A, not applicable; PD‐woNCPs, Parkinson's disease without neurocognitive and psychiatric symptoms; PD‐wNCPs, Parkinson's disease patients with neurocognitive and psychiatric symptoms.

### 2.2 MRI acquisition

A 3T MR scanner (MAGNETOM Prisma; Siemens Healthcare, Erlangen, Germany) with a 64‐channel head coil was used for all imaging. Diffusion‐weighted imaging (DWI) was performed by spin‐echo echo‐planar imaging in the anterior‐to‐posterior phase‐encoding direction. Multishell DWI was performed using two *b* values (1,000 and 2,000 s/mm^2^) along 64 isotropic diffusion gradients for each shell. The sequence parameters were as follows: repetition time (TR), 3,300 ms; echo time (TE), 70 ms; field of view (FOV), 229 × 229 mm; matrix size, 130 × 130; resolution, 1.8 × 1.8 mm; slice thickness, 1.8 mm; and acquisition time, 7.29 min. Acquisition of each diffusion‐weighted image was completed with a gradient‐free image (*b* = 0). Standard and reverse phase‐encoded blipped images with no diffusion weighting (Blip Up and Blip Down) were also acquired to correct for magnetic susceptibility‐induced distortions related to the echo‐planar imaging acquisitions (Andersson & Sotiropoulos, 2016). Three‐dimensional T1‐weighted image magnetization‐prepared 180° radio‐frequency pulses and rapid gradient‐echo sequences were acquired with the following parameters: TR, 2,300 ms; TE, 2.32 ms; inversion time: 900 ms; FOV, 240 × 240 mm; matrix size, 256 × 256; resolution, 0.9 × 0.9 mm; slice thickness, 0.9 mm; and acquisition time, 06.25 min.

### 2.3 Diffusion MRI pre‐processing

The diffusion‐weighted data were corrected for susceptibility‐induced geometric distortions, eddy current distortions, and inter‐volume subject motion using the EDDY and TOPUP toolboxes (Andersson & Sotiropoulos, 2016). We then visually assessed all DWI datasets in the axial, sagittal, and coronal views. All datasets were free from severe artifacts, such as gross geometric distortion, signal dropout, and bulk motion.

The resulting images were fitted to the NODDI model (Zhang et al., 2012) using the NODDI MATLAB Toolbox 5 (http://www.nitrc.org/projects/noddi_toolbox). Maps of ICVF, ODI, and isotropic volume fraction (ISO, Figure S1) were generated using Accelerated Microstructure Imaging via Convex Optimization (Daducci et al., 2015). The diffusion tensor was estimated using ordinary least squares applied to the diffusion‐weighted images with *b* = 0 and 1,000 s/mm^2^. FA, MD, axial diffusivity (AD), and radial diffusivity (RD) maps (Figure S1) were then generated for all subjects using the DTIFIT tool of the FMRIB Software Library 5.0.9 (FSL, Oxford Center for Functional MRI of the Brain, UK; http://www.fmrib.ox.ac.uk/fsl) to fit the tensor model to each voxel of the DWI data (Basser, Mattiello, & LeBihan, 1994).

### 2.4 TBSS analysis

Voxel‐wise statistical analysis was carried out using TBSS (Smith et al., 2006) implemented in FMRIB Software Library version 5.0.9 (FSL; Oxford Centre for Functional MRI of the Brain, Oxford, UK; http://www.fmrib.ox.ac.uk/fsl; Jenkinson, Beckmann, Behrens, Woolrich, & Smith, 2012) to regionally map significant differences in each of the DTI and NODDI indices between groups, as well as to evaluate the relationship between each index and the disease duration or clinical scores, such as MDS‐UPDRS parts I.1–I.6 and III, with the use of a skeleton projection step.

First, we performed nonlinear registration of FA images of all subjects into 1 × 1 × 1 × mm^3^ Montreal Neurological Institute (MNI 152) common space (a normalized/averaged brain) using the FMRIB's nonlinear registration tool (Jenkinson et al., 2012). Second, the transformed FA images were average to create a mean FA image. Third, the mean FA was thinned to create a mean FA skeleton, which represented the centers of all tracts common to the groups. The threshold of the mean FA skeleton was set to >0.20 to include the major WM pathways and exclude the peripheral tracts and GM. The aligned FA map of each subject was then projected onto the skeleton. Finally, the NODDI (ICVF and OD) and DTI (MD, AD, and RD) maps were projected onto the mean FA skeleton after applying the warping registration field of each subject to the standard space.

### 2.5 TOI analysis

Any maps showing significant clusters by TBSS analysis was localized using Johns Hopkins University's ICBM‐DTI‐81 WM tractography atlas, which is composed of 11 structures (forceps major and minor, anterior thalamic radiation [ATR], corticospinal tract [CST], cingulum cingulate gyrus [CCG], cingulum hippocampus [CHp], inferior fronto‐occipital fasciculus [IFOF], inferior and superior longitudinal fasciculus [ILF and SLF, respectively], uncinate fasciculus [UF], and SLF temporal part) (Hua et al., 2008; Wakana et al., 2007). The average diffusion metric was averaged over all WM skeleton voxels comprising a given region, as delineated by the atlas, for all subjects.

### 2.6 Voxel‐based morphometry (VBM)

VBM was used to obtain WM volumetry. Three‐dimensional T1‐weighted images of each subject were segmented into WM, GM, and cerebrospinal fluid with the use of a unified tissue‐segmentation model using Statistical Parametric Mapping (SPM) 12 (Wellcome Department of Imaging Neuroscience, London, UK, http://www.fil.ion.ucl.ac.uk/spm/software/spm12/) run on a Matlab 2014a platform (MathWorks, Natick, MA, USA; https://www.mathworks.com/products/matlab.html; Ashburner & Friston, 2000). We then spatially normalized the segmented WM and GM images to the customized template in the standardized anatomic space with the use of the “Diffeomorphic Anatomical Registration Using Exponentiated Lie Algebra” (DARTEL) algorithm (Ashburner, 2007). To preserve the WM and GM volumes within each voxel, the Jacobian determinants derived from the spatial normalization, acquired with the DARTEL algorithm, and an 8 mm FWHM Gaussian kernel were used to modulate and smooth the images, respectively.

### 2.7 Linked independent component analysis

Data‐driven decomposition of the imaging features obtained from all subjects was classified into independent components using FMRIB's linked independent component analysis (FLICA; http://fsl.fmrib.ox.ac.uk/fsl/fslwiki/FLICA), which models inter‐subject variability across measures, as described in detail in earlier articles (Douaud et al., 2014; Groves et al., 2012). Here, FLICA was conducted with different imaging modalities (DTI [FA, MD, AD, and RD], NODDI [ICVF and ODI], and VBM [WM volume]) with 15 components. The number of components was chosen as recommended elsewhere (http://fsl.fmrib.ox.ac.uk/fsl/fslwiki/FLICA), where the number of components should be less than the number of subjects/4.

### 2.8 Statistical analysis

All statistical analyses were performed using IBM SPSS Statistics for Windows, version 22.0 (IBM Corporation, Armonk, NY, USA), except for general linear model (GLM) analysis, which was performed using the FSL and SPM. A *p* value of <0.05 was considered statistically significant.

The Shapiro–Wilk test was used to assess the normality of the data. The demographic and clinical data were analyzed using the Mann–Whitney U test or unpaired *t*‐test for two groups and one‐way analysis of variance (ANOVA) for three groups for continuous variables and the chi‐squared test for categorical variables.

For TBSS analyses, a voxel‐wise GLM framework with one‐way ANOVA followed by pairwise post hoc comparisons, which included age and sex as covariates, was applied to compare all diffusion metrics among groups (healthy controls vs. PD‐woNCPs vs. PD‐wNCPs) using the FSL randomize tool with 5,000 permutations. The results were then corrected for multiple comparisons by controlling family‐wise error (FWE) and applying threshold‐free cluster enhancement. The same method was applied for voxel‐wise correlation analysis among all diffusion metrics with disease duration or MDS‐UPDRS part III of the whole PD group and with MDS‐UPDRS part I.1–I.6 scores of the PD‐wNCPs group.

For TOI analyses, the mean diffusion metric values in the WM tracts were compared among groups with the Kruskal–Wallis test. Then, the Benjamini–Hochberg false discovery rate (FDR) correction was applied to correct multiple testing (11 TOIs). Pairwise comparisons were then performed to detect significant main effects on any area with significant FDR‐corrected *p* values by conducting nonparametric Mann–Whitney U tests. The mean diffusion metric values of each TOI were then correlated with the disease duration or MDS‐UPDRS part III of the whole PD group and with MDS‐UPDRS part I.1–I.6 scores in PD‐wNCPs using Spearman's rank correlation coefficient. Considering the exploratory nature of this analysis, Bonferroni correction was not applied.

For VBM analysis, WM volumes were compared between groups using GLM analysis of covariance, with age, sex, and total intracranial volume as covariates. Comparisons of WM volumes were corrected for multiple comparisons using the FWE rate.

LICA decomposition was performed using Matlab R2014a software. Permutation testing was used for correction of multiple testing across all LICA components. The subject weights were permuted 10,000 times with respect to group, age, and sex (Doan et al., 2017). Furthermore, differences in the subject loadings of significant LICA components between groups were identified using one‐way ANOVA with least discriminant post hoc analysis. In this study, only one component reached significance; thus, correction for multiple comparisons was not applied. Finally, the effect sizes of pairwise group comparisons in subject loadings of significant LICA components were standardized using Cohen's *d* (Cohen, 1992).

## 3 RESULTS

### 3.1 Study participants

The demographic and clinical details of all groups are summarized in Table 1. There was no significant difference in age and sex among the three groups (healthy controls, PD‐woNCPs, and PD‐wNCPs) or with regard to disease duration, Hoehn and Yahr stage, MDS‐UPDRS part III score, and levodopa equivalent daily dosage between the PD‐woNCPs and PD‐wNCPs groups.

### 3.2 TBSS analysis

**Figure 1:**
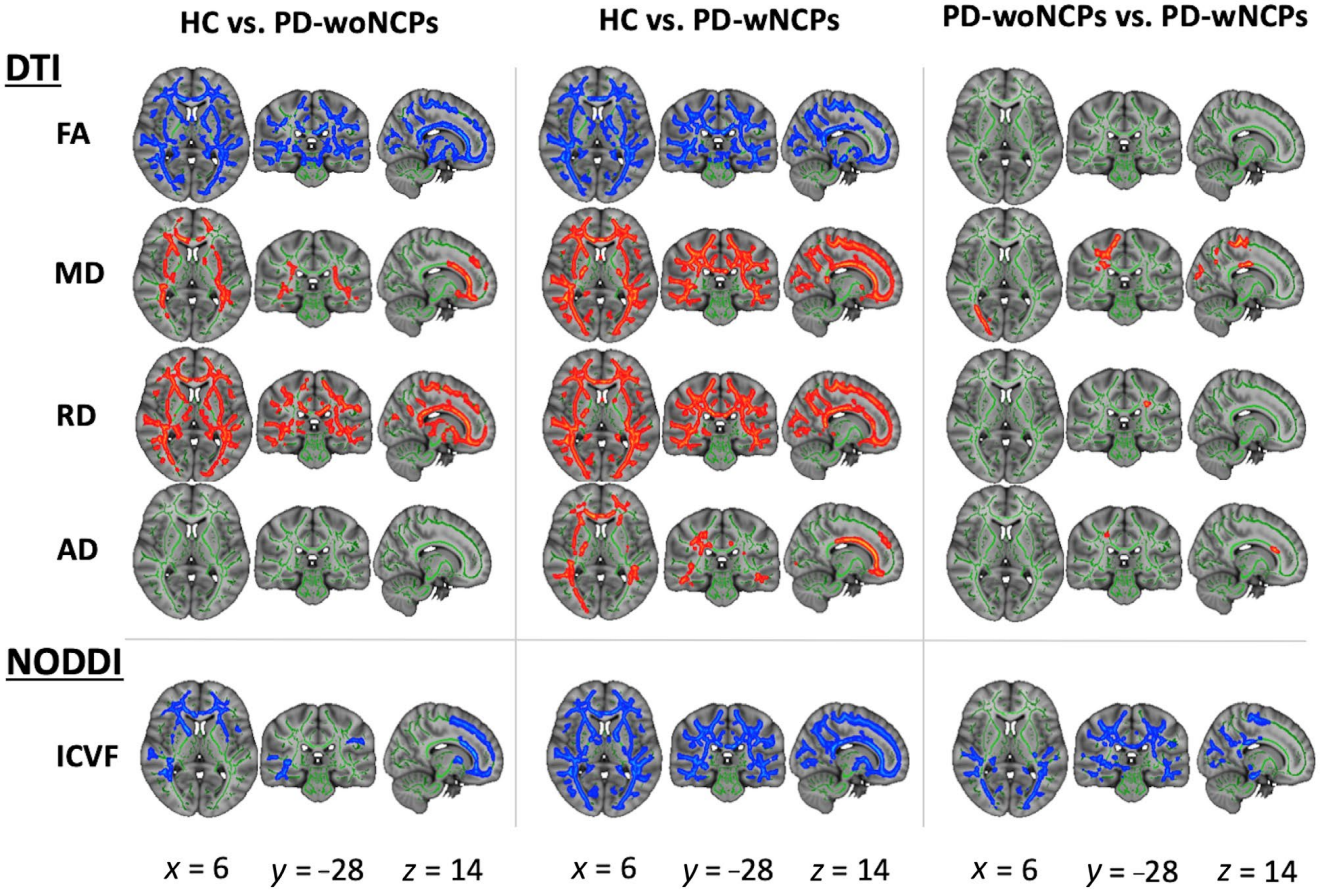
Comparison of DTI (FA, MD, AD, and RD) and NODDI (ICVF) measures among the healthy control, PD‐woNCPs and PD‐wNCPs groups. Using TBSS analysis, significantly lower FA and ICVF (blue–light blue) and significantly higher MD and RD (red–yellow) values were observed in the PD‐woNCPs group compared with the healthy controls. Compared with the healthy controls, the PD‐wNCPs group had significantly lower FA and ICVF and higher MD, RD, and AD values. Compared with the PD‐woNCPs group, the PD‐wNCPs group had significantly lower ICVF and higher MD, RD, and AD values. There were no significant differences in ODI values between the groups. The FA skeleton with FA > 0.2 is shown in green. To aid visualization, results are thickened using the fill script implemented in FMRIB Software Library. Abbreviations: DTI, diffusion tensor imaging; FA, fractional anisotropy; HC, healthy control; ICVF, intracellular volume fraction; MD, mean diffusivity; NODDI, neurite orientation dispersion and imaging; ODI, orientation dispersion index; PD‐woNCPs, Parkinson's disease without neurocognitive–psychiatric symptoms; PD‐wNCPs, Parkinson's disease with neurocognitive and psychiatric symptoms; RD, radial diffusivity [Color figure can be viewed at https://wileyonlinelibrary.com]

Figure 1 and Table 2 show the results of TBSS analysis of the DTI and NODDI indices. Significantly (*p*FWE < 0.05) lower FA and ICVF values and higher MD, RD, and AD values were observed in the PD‐wNCPs group than in the healthy controls, and lower ICVF values and higher MD, RD, and AD values were observed in the PD‐wNCPs group than in the PD‐woNCPs group. Changes in all indices were observed across the broad areas of the WM compared with the healthy controls. Changes in ICVF and MD values predominantly occurred in the posterior portion of the WM, whereas changes in the RD and AD values were observed in the circumscribed areas of the WM compared with the PD‐woNCPs group. Compared with the healthy controls, the PD‐woNCPs group had significantly (*p*FWE < 0.05) lower FA and ICVF values and higher MD and RD values. Changes in the FA and RD values were observed across the broad areas of the WM, whereas changes in the ICVF and MD values predominantly occurred in the anterior portion of the WM. Details regarding the anatomical regions, peak *t*‐values, and peak MNI coordinates of significant clusters are shown in Table 2. There were no significant differences in FA values between the PD‐woNCPs and PD‐wNCPs groups, in AD values between the PD‐woNCPs group and healthy controls, and in ODI values between the PD‐woNCPs group and healthy controls and between PD‐woNCPs and PD‐wNCPs groups.

**Table 2:** Tract‐based spatial statistics analysis of DTI and NODDI indices in patients with Parkinson's disease and healthy controls

Modality	Contrast	Cluster size (number of voxels)	Peak MNI coordinates			Peak *T*‐value	Anatomical region
			X	Y	Z		
*Healthy controls vs. PD‐woNCPs*							
DTI							
FA	HC > PD‐woNCPs	59,368	136	125	95	5.13	Bilateral ATR, CST, CCG, IFOF, ILF, SLF, UF, SLF temporal part, medial lemniscus, ALIC, PLIC, retrolenticular part of internal capsule, ACR, SCR, PCR, PTR, sagittal stratum, external capsule, SFOF, and tapetum; left ICP; right cingulum hippocampus; forceps major and minor, genu, body, and splenium of CC
MD	HC < PD‐woNCPs	14,001	112	156	99	5.1	Bilateral ATR, CST, IFOF, ILF, SLF, UF, SLF temporal part, ALIC, PLIC, retrolenticular part of internal capsule, ACR, SCR, PCR, PTR, sagittal stratum, external capsule, and SFOF; right tapetum; forceps major and minor, genu, body, and splenium of CC
RD	HC < PD‐woNCPs	46,575	135	117	52	5.14	Bilateral ATR, CST, CCG, cingulum hippocampus, IFOF, ILF, SLF, UF, SLF temporal part, ALIC, PLIC, retrolenticular part of internal capsule, ACR, SCR, PCR, PTR, sagittal stratum, external capsule, SFOF, and tapetum; forceps major and minor, genu, body, and splenium of CC
NODDI							
ICVF	HC > PD‐woNCPs	20,403	71	162	78	5.86	Bilateral ATR, CST, CCG, IFOF, ILF, SLF, UF, SLF temporal part, medial lemniscus, ALIC, PLIC, retrolenticular part of internal capsule, ACR, SCR, PCR, PTR, sagittal stratum, external capsule, SFOF, and tapetum; left ICP; right cingulum hippocampus; forceps major and minor, genu, body, and splenium of CC
*Healthy controls vs. PD‐wNCPs*							
DTI							
FA	HC > PD‐wNCPs	74,667	112	162	77	5.86	Bilateral ATR, CST, cingulum hippocampus, IFOF, ILF, SLF, UF, SLF temporal part, cerebral peduncle, ALIC, PLIC, retrolenticular part of internal capsule, ACR, SCR, PCR, PTR, sagittal stratum, external capsule, SFOF, and tapetum; left CCG; forceps major and minor, genu, body, and splenium of CC
MD	HC < PD‐wNCPs	74,615	123	116	35	6.11	Bilateral ATR, CST, CCG, IFOF, ILF, SLF, UF, SLF temporal part, ALIC, PLIC, retrolenticular part of internal capsule, ACR, SCR, PCSR, PTR, sagittal stratum, external capsule, SFOF, and tapetum; right cingulum hippocampus; forceps major and minor, fornix, genu, body, and splenium of CC
RD	HC < PD‐wNCPs	80,304	139	77	67	5.94	Bilateral ATR, CST, CCG, cingulum hippocampus, IFOF, ILF, SLF, UF, SLF temporal part, ALIC, PLIC, retrolenticular part of internal capsule, ACR, SCR, PCR, PTR, sagittal stratum, external capsule, SFOF, and tapetum; forceps major and minor, genu, body, and splenium of CC
AD	HC < PD‐wNCPs	19,781	124	124	46	5.97	Bilateral ATR, CST, IFOF, ILF, SLF, UF, SLF temporal part, ALIC, PLIC, retrolenticular part of internal capsule, ACR, SCR, PCR, PTR, sagittal stratum, external capsule, SFOF; left CCG; right tapetum; forceps major and minor, genu, body, and splenium of CC
NODDI							
ICVF	HC > PD‐wNCPs	96,706	141	93	110	6.34	Bilateral ATR, CST, CCG, cingulum hippocampus, IFOF, ILF, SLF, UF, SLF temporal part, ALIC, PLIC, retrolenticular part of internal capsule, ACR, SCR, PCR, PTR, sagittal stratum, external capsule, SFOF, and tapetum; forceps major and minor, genu, body, and splenium of corpus callosum
*PD‐woNCPs vs. PD‐wNCPs*							
DTI							
MD	PD‐woNCPs < PD‐wNCPs	4,253	61	92	105	4.64	Right CST, CCG, IFOF, ILF, SLF, SLF temporal part, SCR, PCR, PTR, sagittal stratum, forceps major, body and splenium of corpus callosum
RD	PD‐woNCPs < PD‐wNCPs	20	118	97	105	4.93	Left CST, PCR, and SLF
AD	PD‐woNCPs < PD‐wNCPs	146	77	148	93	3.51	Right CST, ACR, and SCR; forceps minor, and genu of CC
NODDI							
ICVF	PD‐woNCPs > PD‐wNCPs	29,790	126	67	66	5.42	Bilateral CST, cingulum hippocampus, IFOF, ILF, SLF, SLF temporal part, retrolenticular part of internal capsule, SCR, PCR, PTR, sagittal stratum, external capsule, tapetum; left PLIC; right ATR, CCG, UF; forceps major, body and splenium of corpus callosum

Abbreviations: ACR, anterior corona radiata; AD, axial diffusivity; ALIC, anterior limb of the internal capsule; ATR, anterior thalamic radiation; CC, corpus callosum; CCG, cingulum cingulate gyrus; CST, corticospinal tract; DTI, diffusion tensor imaging; FA, fractional anisotropy; HC, healthy controls; ICVF, intracellular volume fraction; IFOF, inferior fronto‐occipital fasciculus; ILF, inferior longitudinal fasciculus; MD, mean diffusivity; NODDI, neurite orientation dispersion and density imaging; PCR, posterior corona radiata; PD‐woNCPs, Parkinson's disease without neurocognitive and psychiatric symptoms; PD‐wNCPs, Parkinson's disease patients with neurocognitive and psychiatric symptoms; PLIC, posterior limb of the internal capsule; PTR, posterior thalamic radiation; RD, radial diffusivity; SCR, superior corona radiata; SFOF, superior fronto‐occipital fasciculus; SLF, superior longitudinal fasciculus; UF, uncinated fasciculus.

### 3.3 TOI analysis

**Figure 2:**
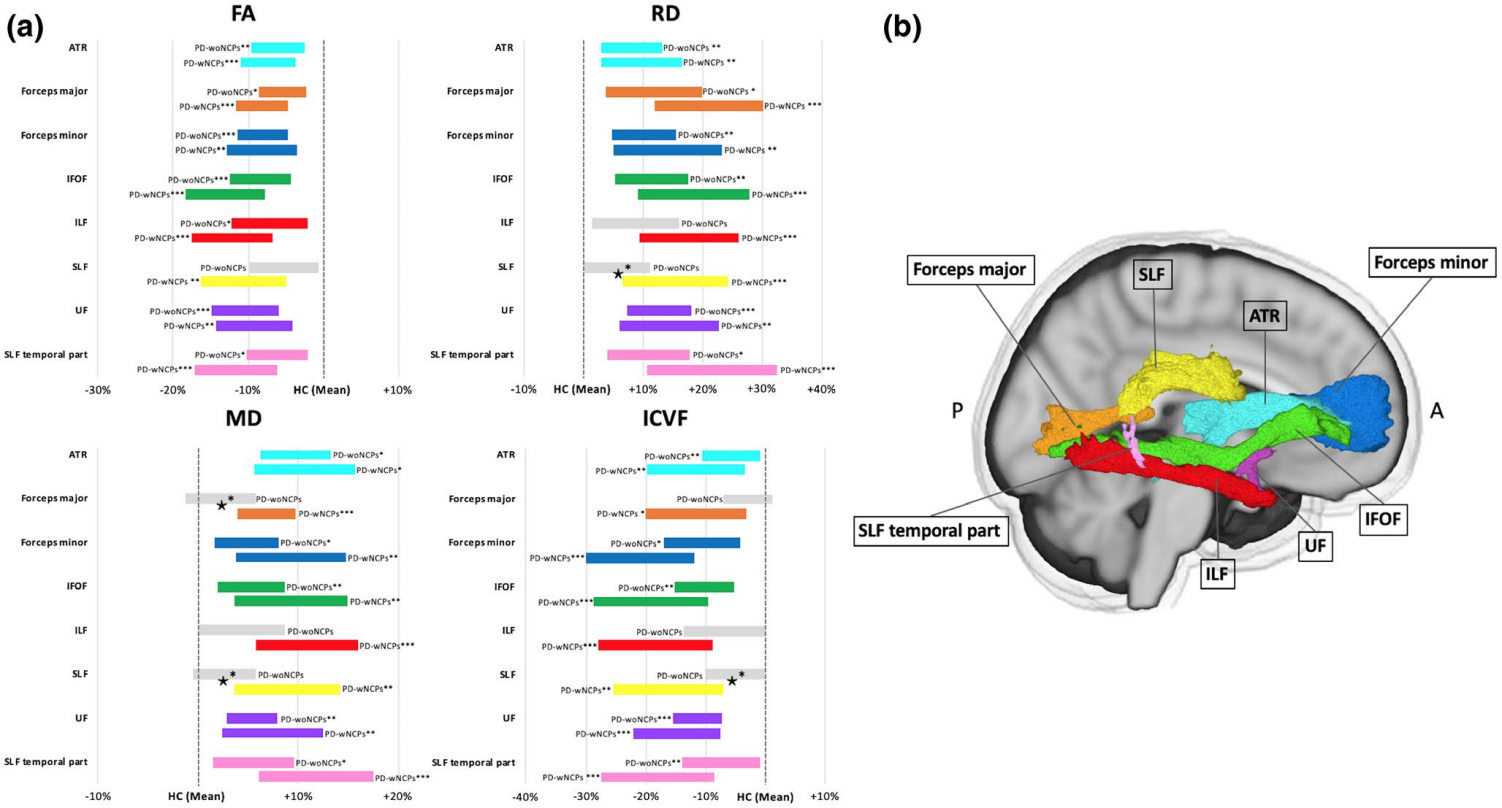
Significant tracts from tract‐of‐interest analysis comparing diagnostic groups. (a) Mean of each measure in the PD‐woNCPs and PD‐wNCPs groups (represented as the percentage difference from the healthy controls). Significant tracts (*p < .05, **p < .01, ***p < .001) are displayed in color, whereas non‐significant tracts are shown in gray. ^★^Tracts with significant differences between the PD‐woNCPs and PD‐wNCPs groups. (b) Tracts obtained using the ICBM‐DTI‐81 white matter tractography atlas. Abbreviations: ATR, anterior thalamic radiation; FA, fractional anisotropy; HC, healthy control; ICVF, intracellular volume fraction; IFOF, inferior fronto‐occipital fasciculus; ILF, inferior longitudinal fasciculus; MD, mean diffusivity; PD‐woNCPs, Parkinson's disease without neurocognitive and psychiatric symptoms; PD‐wNCPs, Parkinson's disease with neurocognitive and psychiatric symptoms; RD, radial diffusivity; SLF, superior longitudinal fasciculus; UF, uncinate fasciculus [Color figure can be viewed at https://wileyonlinelibrary.com]

Figure 2 shows the TOI analysis results for the DTI (FA, MD, RD, and AD) and NODDI (ICVF) indices. As compared with the healthy controls, the PD‐woNCPs group had significantly (*p* < .05) lower FA (ATR, forceps major and minor, IFOF, ILF, SLF temporal part, and UF) and ICVF (ATR, forceps minor, IFOF, SLF temporal part, and UF) values and higher MD (ATR, forceps minor, IFOF, ILF, SLF temporal part, and UF) and RD (ATR, forceps major and minor, IFOF, SLF temporal part, and UF) values. Furthermore, the PD‐wNCPs group had lower FA and ICVF values and higher MD and RD (ATR, forceps major and minor, IFOF, ILF, SLF, SLF temporal part, and UF) values, as compared with the healthy controls. As compared with the PD‐woNCPs group, the MD (forceps major, and SLF), RD (SLF), and ICVF (SLF) values were lower in the PD‐wNCPs group.

### 3.4 Voxel‐based morphometry

There was no significant difference in total and focal WM volumes among the three groups.

### 3.5 Linked independent component analysis

**Figure 3:**
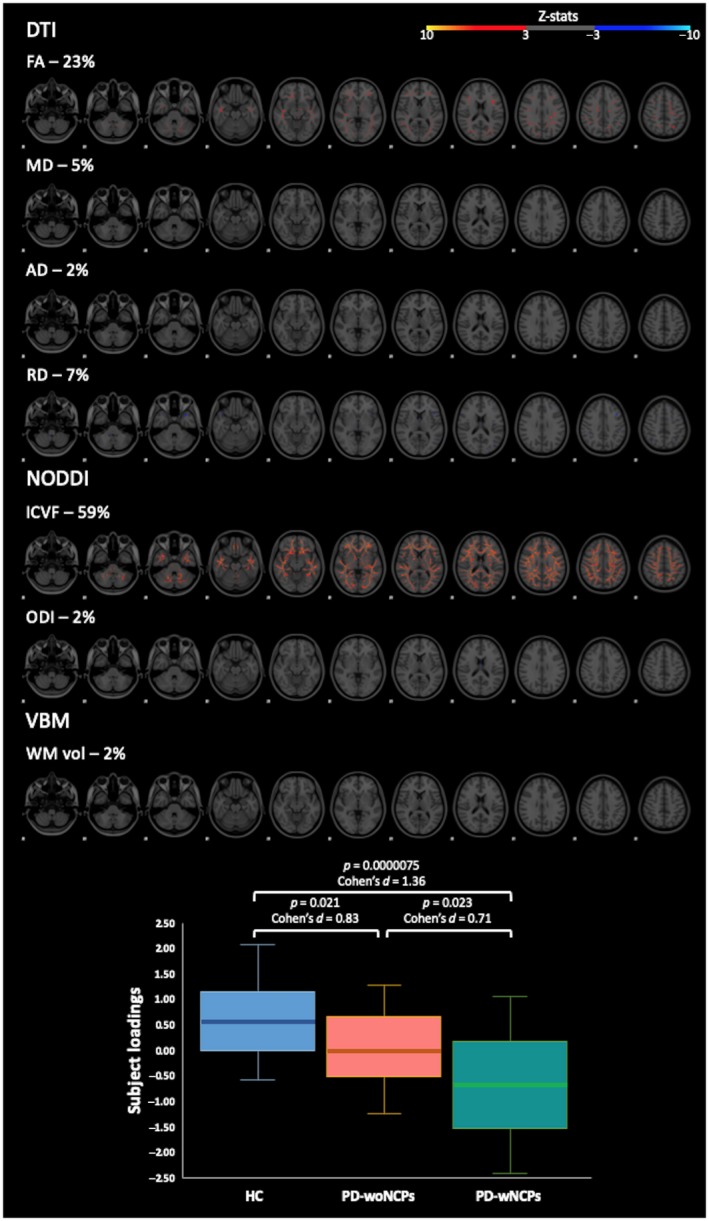
*Upper panel*: Spatial maps of independent component #1. The spatial maps represent the thresholded *z*‐scores (3 < |*z*| < 10). In the spatial maps, the weights (in percentage) indicate the relative contribution of each measure to the component at the group level. *Lower panel*: Boxplot of subject loadings for the three different groups. The bottom and top of the box are first and third quartiles, and the thick band inside the box is the median. Whiskers represent maximum and minimum values of all data. Abbreviations: DTI, diffusion tensor imaging; FA, fractional anisotropy; HC, healthy control; ICVF, intracellular volume fraction; MD, mean diffusivity; NODDI, neurite orientation dispersion and imaging; ODI, orientation dispersion index; PD‐woNCPs, Parkinson's disease without neurocognitive and psychiatric symptoms; PD‐wNCPs, Parkinson's disease with neurocognitive and psychiatric symptoms; RD, radial diffusivity; VBM, voxel‐based morphometry; WM vol, WM volume [Color figure can be viewed at https://wileyonlinelibrary.com]

One component (independent component #1 [IC1]) had significant (*F* = 12.03; corrected *p* < .05) main effects on the diagnosis (Figure S2). IC1 is a multimodal component mainly driven by ICVF (59% weight) followed by FA (23%), RD (7%), MD (5%), AD (2%), ODI (2%), and WM volume (2%) (Figure 3). The PD‐wNCPs group had significantly weaker subject loadings than the healthy controls (*p* = .0000075; Cohen's *d* = 1.36) and PD‐woNCPs (*p* = .023, Cohen's *d *= 0.71) group. The PD‐woNCPs group also had significantly weaker subject loadings than those of the healthy controls (*p* = .021, Cohen's *d* = 0.83) (Figure 3). As indicated in Figure 3, weaker subject loadings in the PD‐wNCPs group reflected widespread decreases of ICVF in the WM.

### 3.6 Correlation analysis

There were no significant correlations between all metrics in all analyses and disease duration or MDS‐UPDRS part III in the whole PD group and MDS‐UPDRS part I.1‐I.6 in the PD‐wNCPs group.

## 4 DISCUSSION

In the present study, NODDI, a novel technique for analyzing multishell DWI data, was applied to investigate microstructural changes in the WM related to the NCPs of PD patients. The major findings of this study were that (a) compared to DTI, NODDI gave a pattern of results that was more regionally specific, (b) both TBSS and TOI analyses showed axonal loss in the WM of PD patients with and without NCPs, whereas the PD‐wNCPs group had more extensive axonal pathology (posterior predominant, especially the SLF), as compared with the PD‐woNCPs group, and (c) in LICA, ICVF, the measure of neurite density had the highest contributions to the main effect of diagnosis.

Lower ICVF in the WM areas of PD patients with and without NCPs, which indicated an axonal density reduction (Zhang et al., 2012), support the view that axonal pathology is a major factor underlying microstructural changes in the WM of PD patients. Indeed, the accumulation of α‐synuclein in PD has been shown to begin in the axonal compartment and is closely associated with axonal degeneration (Chung, Koprich, Siddiqi, & Isacson, 2009; Kamagata et al., 2017).

The anterior brain has been implicated as a region that is more prone to Lewy pathology than the posterior brain (Cochrane & Ebmeier, 2013; Luk & Lee, 2014). Similarly, visualization of the TBSS results showed that axonal loss occurred predominantly in the anterior aspect of the brain of the PD‐woNCPs group, as compared with that of the healthy controls. This finding is consistent with that of a previous meta‐analysis showing that the changes in diffusion measures occur consistently in the frontal lobe of PD patients (Cochrane & Ebmeier, 2013).

Notably, axonal pathology occurred throughout the WM and predominantly in the posterior aspect of the brain (such as forceps major, body, and splenium of corpus callosum, cingulum, posterior limb of internal capsule, posterior corona radiata, posterior thalamic radiation, and some long association fibers) in the PD‐wNCPs group, as compared with that of the healthy controls and PD‐woNCPs group, respectively. Our results are in agreement with those of previous DTI studies showing a lower FA value in the anterior cingulate fiber tracts of PD patients without dementia and in the anterior and posterior cingulate fiber tracts of PD patients with dementia, as compared with that of healthy controls, and in the posterior cingulate fiber tracts of PD patients with dementia, as compared with those without (Kamagata et al., 2012; Matsui et al., 2007). These findings also support the fact that NCPs might manifest to different extents within individual PD patients (Titova, Padmakumar, Lewis, & Chaudhuri, 2017). Although the exact pathological distribution in the WM is not well established, the NCPs of PD patients have been linked to the widespread distribution of degenerated axons (Jellinger, 2011). Furthermore, the density and distribution of Lewy body pathology were shown to influence the severity of NCPs (Apaydin, Ahlskog, Parisi, Boeve, & Dickson, 2002). Accordingly, our results suggest that the posterior aspect of the brain plays some important roles in the progression of the NCPs of PD patients. These results are in line with the findings of some resting‐state functional MRI studies, which have demonstrated alterations in resting‐state brain activity in the default mode network that consists of the precuneus/posterior cingulate cortex; medial prefrontal cortex; and medial, lateral, and inferior parietal cortex in PD patients with NCPs (Hu et al., 2015; Jia, Li, Li, Liang, & Fu, 2018; Shin et al., 2017; van Eimeren, Monchi, Ballanger, & Strafella, 2009). The fact that PD pathology progresses from the anterior to the posterior parts of the WM (Braak & Del Tredici, 2008; Brundin, Ma, & Kordower, 2016) partially explains the findings of this study.

Axonal loss in the WM tracts implicated in PD‐wNCPs was largely congruent with the regions of WM abnormalities reported in previous DTI studies. Changes in DTI measures, such as lower FA or higher MD values, have been demonstrated in the ATR, anterior and superior corona radiata, CST, ILF, IFOF, SLF, UF, cingulum, corpus callosum, forceps major and minor, and limb of internal capsule of PD patients with cognitive impairment (Agosta et al., 2014; Hattori et al., 2012; Kamagata et al., 2012, 2013; Rae et al., 2012); in the ATR, ILF, SLF, UF, and forceps minor of PD patients with depression (Huang et al., 2014); in the ATR, SLF, UF, and cingulum of PD patients with apathy (Lucas‐Jimenez et al., 2018); in the hippocampus of PD patients with hallucinations (Yao et al., 2016); and in the CST, ILF, and corpus callosum of PD patients with impulse control disorders (Mojtahed Zadeh, Ashraf‐Ganjouei, Ghazi Sherbaf, Haghshomar, & Aarabi, 2018). Furthermore, in TOI analysis, WM microstructural changes (i.e., lower ICVF and higher MD and RD) in PD‐wNCPs, as compared with those in PD‐woNCPs, were consistently found in the SLF by TOI analysis. This finding suggests that NCPs in PD is related to impaired long WM nerve fibers, especially the SLF. SLF is an association fiber bundle connecting the frontal, occipital, parietal, and temporal lobes and is closely related to the functions of the frontal lobe (Jiang, Shi, Niu, Xie, & Yu, 2015). Indeed, the NCPs of PD patients have been linked to the impairment of multiple neurotransmitter (dopaminergic Biundo, Weis, & Antonini, 2016; Schapira et al., 2017), noradrenergic (Delaville, Deurwaerdere, & Benazzouz, 2011; Schapira et al., 2017), and cholinergic (Nagasaka, Watanabe, & Takashima, 2017; Perez‐Lloret & Barrantes, 2016) pathways that are projected to the frontal lobe. Furthermore, previous studies have consistently showed alterations in the diffusion metrics in the SLF of PD patients with cognitive impairment (Hattori et al., 2012; Kamagata et al., 2012, 2013), apathy (Lucas‐Jimenez et al., 2018), and depression (Huang et al., 2014).

Here, comprehensive analysis was also performed by fusing data of the measured indices (DTI [FA, MD, AD, and RD], NODDI [ICVF and ODI], and VBM [WM volume]) using LICA (Groves et al., 2011, 2012). LICA is a probabilistic technique based on a Bayesian framework that provides an effective way for simultaneously modeling covariances across modalities (Groves et al., 2011, 2012). By decomposing the imaging data into a set of independent components, LICA enables an integrated perspective that may improve clinical sensitivity, as compared with unimodal analyses. In the current study, the only component of LICA that significantly contributed to the main effects of the diagnosis of the NCPs of PD patients and explained the covariance patterns of all included metrics was the ICVF (59% weight). Furthermore, corresponding to the results of TBSS and TOI analyses, a global pattern of decreasing ICVF was shown with advancing NCPs, where PD‐wNCPs exhibited the weakest subject loadings, followed by PD‐woNCPs and healthy controls with widespread decreases of ICVF in the WM. Together, these results suggest that ICVF is a useful measure of group differences and a biomarker for the detection of microstructural changes in the WM related to the NCPs of patients with PD.

Increased MD, an index of increased accumulation of water content and spacing between membrane layers due to neuronal loss (Zhang et al., 2012), in WM tracts had overlapped, at least to some extent, with those displaying lower ICVF. MD was shown to be more accurate than FA (Wiltshire et al., 2010), and a recent investigation in patients with premanifest Huntington's disease using NODDI also showed widespread reduction in ICVF that overlapped with increased MD (Zhang et al., 2018). A strong negative correlation has been demonstrated between the ICVF and MD maps in the human brain (Fukutomi et al., 2018). On the contrary, other DTI indices have been shown to be inconsistent. For example, in TBSS and TOI analyses, the PD‐woNCPs and PD‐wNCPs groups had lower FA and higher RD values in broad areas of the WM, as compared with the healthy controls. FA and AD failed to demonstrate differences between the PD‐woNCPs and PD‐wNCPs groups and between the healthy control and PD‐woNCPs groups, respectively. Likewise, the studies by Kamagata et al. (2012) and Melzer et al. (2013) also failed to show the differences between PD with versus. without dementia or between PD with mild cognitive impairment versus. PD with dementia, respectively. DTI indices are reportedly sensitive, but not specific, to microstructural changes (Andersson & Sotiropoulos, 2016). Decreases in FA may arise not only from the axonal loss but also from demyelination and changes in the size of axons (Hall et al., 2016). Furthermore, AD and RD may provide an acceptable approximation if the voxel includes a healthy fiber bundle determining the diffusion characteristic of the voxel. If the signal‐to‐noise ratio is low, if crossing fibers are present, or if pathology causes a decrease in anisotropy, such an approach can lead to misinterpretation of the results (Wheeler‐Kingshott, Ciccarelli, Schneider, Alexander, & Cercignani, 2012).

There were some limitations to this study. First, this was a cross‐sectional study with a relatively small sample size. Multicenter longitudinal studies with larger sample sizes are still needed. Furthermore, this study included PD patients with relatively long disease durations (>9 years). Because the NCPSs of PD patients might occur earlier than motor symptoms (Schapira et al., 2017), a study of patients in the prodromal stage of PD is required to determine whether NODDI is sufficiently sensitive to reflect the pathology in the WM related to the NCPSs of PD patients. Second, this study combined six domains of NCPSs of PD patients. Even though each domain of NCPSs might have overlapping pathophysiology and some symptoms might be similar, studies on each domain might be useful to reveal the specific pathology. The heterogeneity of NCPSs might also be the cause, as there was no correlation between the DWI metrics and MDS‐UPDRS parts I.1–I.6. However, widespread axonal degeneration was demonstrated in PD patients with MDS‐UPDRS parts I.1–I.6 more than 1, indicating that the scale might be suitable for the detection of the NCPs of PD patients. Third, the clinical scores to assess NPS in this study were limited to MDS‐UPDRS part I, and there are many other clinical scores that are more specific to each domain (Huang et al., 2014). Furthermore, although we only included PD patients with no significant difference in MDS‐UPDRS part III and Hoehn and Yahr score, the presence of motor symptoms may have interfered with the ability to evaluate non‐motor abnormalities (Schapira et al., 2017). Finally, in this study, we only obtained one *b* = 0 image; thus, it is not possible to correct the signal drift, which causes a global signal decrease with subsequently acquired images. This might have affected the estimation of the diffusion parameters (Vos et al., 2017).

## 5 CONCLUSIONS

The results of this study support the view that axonal loss is a major factor underlying NCPSs‐related WM microstructural changes in PD. Decreased axonal density, as a result of α‐synuclein deposition and multiple neurotransmitter deficiencies, was found to be broader (posterior predominant) in the PD‐wNCPs group than in the PD‐woNCPs group. Furthermore, the LICA results showed that ICVF can be used as a useful measure for the evaluation of microstructural changes in the WM related to the NCPs of patients with PD. Taken together, these results suggest the potential of NODDI as an imaging biomarker for NCPs‐related microstructural changes in the WM of PD patients.

## DECLARATION OF TRANSPARENCY

The authors, reviewers, and editors affirm that in accordance with the policies set by the *Journal of Neuroscience Research* this manuscript presents an accurate and transparent account of the study being reported and that all critical details describing the methods and results are present.

## CONFLICT OF INTEREST

The authors have no conflict of interest to declare.

## AUTHOR CONTRIBUTIONS

*Conceptualization*, C.A., K.K., T.H., T.O., H.T.‐A., N.H., and S.A.; *Data Curation*, C.A., Yu.S., W.U., S.M., G.O., Ya.S., A.U., and N.H.; *Formal Analysis*, C.A.; *Investigation*, C.A. and A.H.; *Methodology*, C.A., K.K., T.H., Yu.S., W.U., T.O., H.T.‐A., and S.A.; *Writing – Original Draft*, C.A.; *Writing – Review & Editing*, C.A., K.K., T.H., T.O., H.T.‐A., A.H., G.O., Ya.S., A.U., T.A., A.W., K.K.K., M.H., N.H., and S.A.; *Funding Acquisition*, K.K. and S.A.; *Supervision*, K.K., T.H., T.A., A.W., K.K.K., M.H., and S.A.; *Validation*, K.K., A.H., and M.H.; *Software*, Yu.S. and W.U.; *Visualization*, Yu.S., W.U., and S.M.

## Supporting information


**Figure S1** DTI (FA, MD, AD, and RD) and NODDI (ICVF, ODI, and ISO) maps of one healthy control and one patient with Parkinson's disease. Abbreviations: AD, axial diffusivity; FA, fractional anisotropy; ICVF, intracellular volume fraction; ISO, isotropic volume fraction; MD, mean diffusivity; ODI, orientation, dispersion index; RD, radial diffusivity
**Figure S2** Relative weights indicating the contribution of each modality within each component

Transparent Science Questionnaire for Authors

Transparent Peer Review Report

## Data Availability

The data that support the findings of this study are available from the corresponding author upon reasonable request.
