# Supplementary material for: Neurocognitive and psychiatric disorders‐related axonal degeneration in Parkinson's disease
Source: J Neurosci Res. 2020 Feb 5;98(5):936–49. doi: 10.1002/jnr.24584 (PMC7154645; doi:10.1002/jnr.24584)
Supplement: Supplementary file 1 — Figure S1 DTI (FA, MD, AD, and RD) and NODDI (ICVF, ODI, and ISO) maps of one healthy control and one patient with Parkinson's disease. Abbreviations: AD, axial diffusivity; FA, fractional anisotropy; ICVF, intracellular volume fraction; ISO, isotropic volume fraction; MD, mean diffusivity; ODI, orientation, dispersion index; RD, radial diffusivity Figure S2 Relative weights indicating the contribution of each modality within each component [file JNR-98-936-s001.docx]

**Supplementary figures**

**
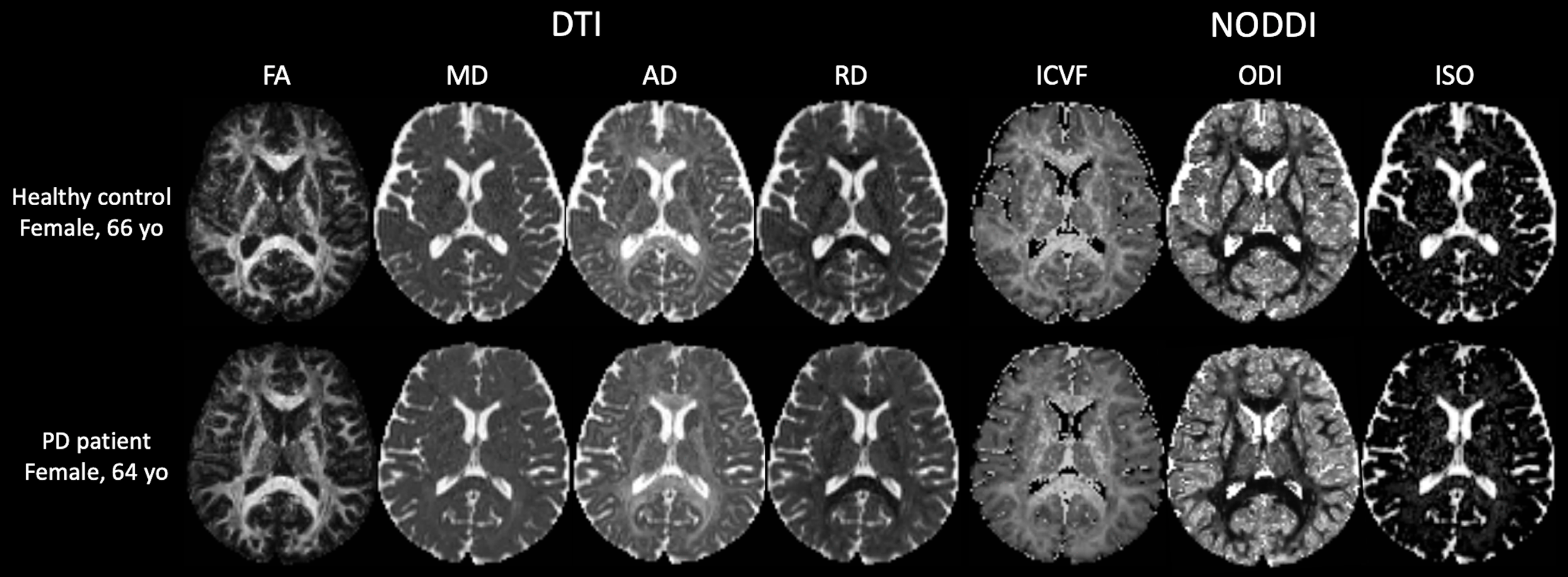
**

**Supplementary Figure 1.** DTI (FA, MD, AD, and RD) and NODDI (ICVF, ODI, and ISO) maps of one healthy control and one patient with Parkinson’s disease. AD, axial diffusivity; FA, fractional anisotropy; ICVF, intracellular volume fraction; MD, mean diffusivity; ISO, isotropic volume fraction; ODI, orientation, dispersion index; RD, radial diffusivity.


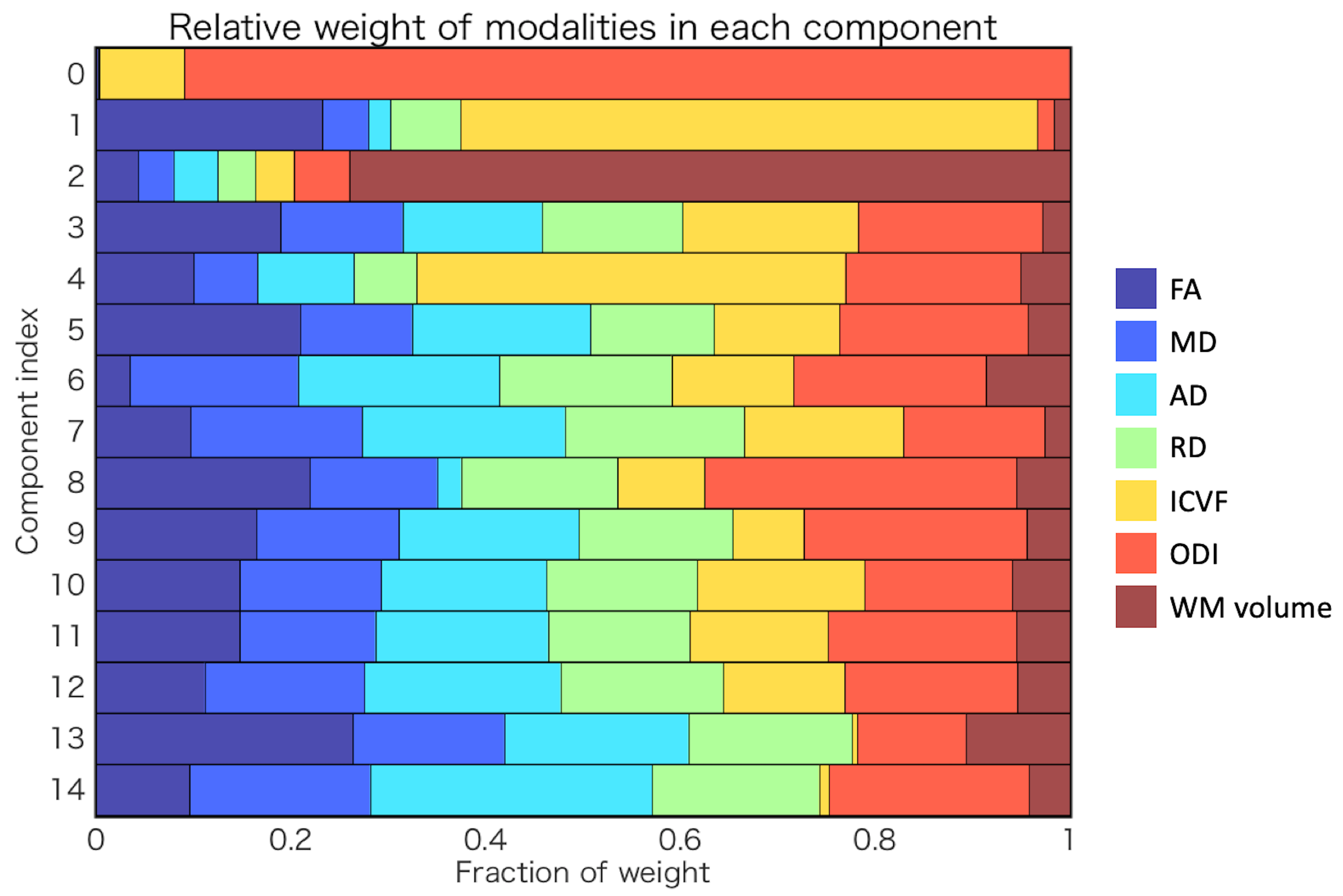


**Supplementary Figure 2.** Relative weights indicating the contribution of each modality within each component.
