# Supplementary material for: Neurocognitive and psychiatric disorders‐related axonal degeneration in Parkinson's disease
Source: J Neurosci Res. 2020 Feb 5;98(5):936–49. doi: 10.1002/jnr.24584 (PMC7154645; doi:10.1002/jnr.24584)
Supplement: Supplementary file 3 — Transparent Peer Review Report [file JNR-98-936-s003.docx]

Neurocognitive and psychiatric disorders-related axonal degeneration in Parkinson's disease

Andica, Christina; Kamagata, Koji; Hatano, Taku; Uchida, Wataru; Ogawa, Takashi; Takeshige-Amano, Haruka; Hagiwara, Akifumi; Murata, Syo; Oyama, Genko; Shimo, Yashushi; Umemura, Atsushi; Akashi, Toshiaki; Wada, Akihiko; Kumamaru, Kanako; Hori, Masaaki; Hattori, Nobutaka; Aoki, Shigeki

_____________________________________________________________________________________

| Review timeline: | Submission date: 24/10/2019  Editorial Decision: Minor Modification  Revision Received: 05/12/2019  Accepted: 06/01/2020 |  |
| --- | --- | --- |
|  |  |  |

Editor: Sandra Chanraud

Reviewer : Bryce Geeraert

Reviewer : Henrik Sjostrom

_____________________________________________________________________________________

1st Editorial Decision	21/11/19

_____________________________________________________________________________________

Dear Dr Andica:

Thank you for submitting your manuscript to the Journal of Neuroscience Research. We do apologize for the delay getting the manuscript and comments back to you. We finally have received the reviewers feedback and have appended them below. The reviewers have appreciated reading your manuscript but as you will read, they have minor concerns and they do not find the manuscript suitable for publication in its current form. One concern highlighted by the reviewers is that the diffusion acquisition has to be more detailed. In the same way, findings have to be deepen and clarified. In agreement with reviewers comments, we do recommend minor revisions before considering the manuscript for publication in JNR.

We ask that you return your manuscript within 30 days. Please explain in your cover letter how you have changed the present version. If you require longer than 30 days to make the revisions, please contact Dr. Eric Prager (eprager@wiley.com). You can submit your revised manuscript directly by clicking on the following link: *** PLEASE NOTE: This is a two-step process. After clicking on the link, you will be directed to a webpage to confirm. ***

https://mc.manuscriptcentral.com/jnr?URL_MASK=1797523efe1f4de4a4cfd16dbe7f6cdb

Thank you again for your submission to the Journal of Neuroscience Research; we look forward to reading your revised manuscript.

Best Wishes,

Dr Sandra Chanraud

Associate Editor, Journal of Neuroscience Research

Dr Eric Prager

Editor-in-Chief, Journal of Neuroscience Research

Reviewer: 1

Comments to the Author

This project applies two diffusion-based imaging techniques, DTI and NODDI, to investigate white matter alterations in Parkinson’s disease. Subjects were compared between healthy controls (n = 20) and Parkinson’s disease patients with (n = 20) and without (n = 19) neurocognitive and psychiatric disorders. Using TBSS, this project showed global reductions in intracellular volume fraction (ICVF) in Parkinson’s disease patients, with greater emphasis on posterior pathology in patients with neurocognitive and psychiatric disorders. One component of a linked ICA analysis was discussed. This component was driven by ICVF (59% weight) and had a main effect for diagnosis, such that healthy controls had the highest loadings, and Parkinson’s disease patients with neurocognitive and psychiatric disorders had the lowest weightings of this component. Overall, this project is very well designed and informative to the field, although increased clarity is needed in some areas of the manuscript, particularly in the Methods section.

Note that page numbers for these comments are based on numbers listed at the top of the pages.

Page 4, line 55: “…intracellular volume fraction, the parameter of neurite orientation…”: As written this sentence may lead a reader to think that intracellular volume fraction is the only useful parameter from the NODDI technique, but ODI and isotropic volume fraction are important NODDI parameters as well. I suggest rephrasing to avoid this implication.

Page 6, line 17: “…packing density of axons in WM and spatial organization of the axons, respectively, which are two separate disentangled facets of FA…”: Separate and disentangled serve as redundant terms to each other. Remove one of these terms to improve clarity.

Page 7, section 2.2:

- Line 38: “…64 isotropic diffusion gradients in the anterior-posterior phase-encoding direction…”: Is this worded correctly? All diffusion gradients chosen for each diffusion-weighted volume in the image were oriented in the same direction? Also, were 64 directions collected for b = 1000 and b = 2000 each, or 32 directions each resulting in 64 total?

- Line 43: DWI is used as an acronym here inappropriately, as it is defined as Diffusion Weighted Imaging above in line 34.

- Line 43: Does this sentence imply that after 64 diffusion directions, one b0 image is required total? If only one b0 image was collected, authors should address the inability to correct for signal drift throughout their diffusion sequences as a limitation to the accuracy of their diffusion-based measures (see Vos et al., 2016, https://doi.org/10.1002/mrm.26124)

Page 8, section 2.3:

- Line 10: “…each of 64 different axial, sagittal, and coronal directions.”: This sentence did not help to clarify my understanding of the diffusion directions chosen. To this reviewer, axial, sagittal, and coronal refer to three specific planes of anatomical orientation. How are the 64 “directions” oriented within these 3 planes, and how does this reconcile with the previous statement (page 3 line 38) that all 64 directions were isotropic in the anterior-posterior direction?

- Paragraph 2: Regarding NODDI measure quality checking and interpretation, did the authors review isotropic volume fraction (fiso) images to ensure the anatomical relevance of their ICVF and ODI maps? Essentially, if fiso is high in a region, ICVF and ODI values are arbitrary. See this discussion in the NODDI google group for a starting point on why ICVF and ODI should not be interpreted in absence of fiso interpretation: https://groups.google.com/forum/#!searchin/noddi/high$20ficvf%7Csort:date/noddi/OhXUUzBpMHI/9YK5C8isykkJ

Page 11, line 53: “…no significant differences in AD or ODI values between…”: I thought it would be helpful to add a similar line to the caption of Figure 1, as I flipped back to this section to double check why ODI was not shown once I had viewed Figure 1.

Page 11 & 12, sections 3.2.1, 3.2.2, 3.2.3: I found it odd that several sentences were repeated word for word between sections. I would prefer these sections to be combined into one section to reduce repetition.

Page 13, section 3.5: What did other components from the linked ICA look like? Was ICVF present in multiple components or was it limited to IC1? Inclusion of a figure visualizing the makeup of components output by linked ICA would serve as a useful reference for future studies replicating or extending the work presented here.

Page 13, line 42: “…the measure of neurite density had the highest contributions to the main effect of diagnosis.”: Did the authors consider running follow up analysis with ICVF alone to augment their interpretation of ICVF as a “sensitive biomarker to detect microstructural changes in white matter related to neurocognitive and psychiatric disorders in Parkinson’s disease.” ?

Minor comments:

- The authors should consider a visualization of their raw data to give readers a reference on how each measure presents in healthy and Parkinson’s disease patients, and to aid in the reader’s assessment of overall data quality.

Reviewer: 2

Comments to the Author

The authors have investigated microstructural white matter alterations measured by diffusion weighted imaging and their association to neurocognitive symptoms in PD, compared to PD without neurocognitive symptoms and healthy controls. The paper is well-written, and the authors describe white matter changes linked to cognitive abnormalities, where intra-cellular volume fraction seems to show the highest contribution to the found abnormalities. The writing is concise and overall relavant, but I do have some concerns.

-In the abstract, you state that you included 20 and 19 PD patients with and without NCPs and 20 healthy controls. However, in the methods and in Table 1, you write 25 healthy healthy controls. Which is correct?

-In multiple places, you state that your results suggest that ICV can be used as a sensitive biomarker to detect microstructural changes. Have you tested this sensitivity in detection of microstructural changes in this paper? If you have not, I suggest that you change this wording. You also say in the discussion (page 16), that “…a study of patients in the prodromal stage of PD is required to determine whether NODDI is sufficiently sensitive to reflect the pathology in the WM related to the NCPs of PD patients”, which contradicts your above statement.

-On page 6, lines 10-13, you summarize studies demonstrating superiority of NODDI over DTI. In this summary, you mention a study by Surova et al, which actually uses another variant of neurite density imaging. So despite being relatively similar to NODDI, it is not really true that it demonstrates NODDI superiority over DTI. It is however a relavant study to mention in your paper, but maybe with some rephrasing.

-In table 1 you report H&Y score as mean ± SD. This is inappropriate since H&Y is an ordinal variable with few levels. Please change to reporting frequencies (N/N/N/N/N). The statistic test should for testing differences in H&Y should then also be changed to chi-square.

-In “2.2 MRI acquisition” you also say “Acquisition of each DWI was completed at b = 0 with no diffusion gradient.” This is unclear and should be rephrased. How many b = 0 volumes were acquired, and how were they distributed? Were they interspersed?

In “2.4 TBSS analysis” you state that “First, FA images of all subjects were affine-transformed into 1 x 1 x 1 mm3…” “…using the FMRIB’s nonlinear registration tool…”. Affine means linear, and can not be done with FNIRT, so some correction is needed here.

In page 9, line 3, you state “Without initial registration, the maps were projected onto the mean FA skeleton”. Do you really project maps from individual subjects onto a mean FA skeleton without any prior registration? This is unclear and needs to be better explained.

In “2.8 Statistical analysis”, the TBSS section only mentions ANOVA techniques, but from your results, it seems that pairwise comparisons were also conducted from the TBSS (sections 3.2.2 and 3.2.3). This needs to be clearer.

____________________________________________________________________________________

Authors’ Response	05/12/19

_____________________________________________________________________________________

We would like to thank the reviewers for their thoughtful suggestions and insights, which have helped us to improve our manuscript and present our research in a better and balanced manner. Our specific responses to the reviewers’ comments are provided below. The deleted text is indicated using strikethrough, and the revisions are highlighted in yellow in the revised manuscript.

Reviewer: 1

Comments to the Author
This project applies two diffusion-based imaging techniques, DTI and NODDI, to investigate white matter alterations in Parkinson’s disease. Subjects were compared between healthy controls (n = 20) and Parkinson’s disease patients with (n = 20) and without (n = 19) neurocognitive and psychiatric disorders. Using TBSS, this project showed global reductions in intracellular volume fraction (ICVF) in Parkinson’s disease patients, with greater emphasis on posterior pathology in patients with neurocognitive and psychiatric disorders. One component of a linked ICA analysis was discussed. This component was driven by ICVF (59% weight) and had a main effect for diagnosis, such that healthy controls had the highest loadings, and Parkinson’s disease patients with neurocognitive and psychiatric disorders had the lowest weightings of this component. Overall, this project is very well designed and informative to the field, although increased clarity is needed in some areas of the manuscript, particularly in the Methods section.

Note that page numbers for these comments are based on numbers listed at the top of the pages.

Page 4, line 55: “…intracellular volume fraction, the parameter of neurite orientation…”: As written this sentence may lead a reader to think that intracellular volume fraction is the only useful parameter from the NODDI technique, but ODI and isotropic volume fraction are important NODDI parameters as well. I suggest rephrasing to avoid this implication.

**Response:** According to your suggestion, we have revised the significance statement, as follows:

**Significance Statement:** (Page 3, Line 26 – Page 4, Line 2)

Using neurite orientation dispersion and density imaging, this study demonstrated that axonal loss, indicated by a reduction in the intracellular volume fraction, is a major factor underlying white matter pathology related to neurocognitive and psychiatric disorders in Parkinson's disease. However, patients with neurocognitive and psychiatric disorders had broader axonal pathology (posterior predominant) compared with those without such disorders. Linked independent component analysis suggested that intracellular volume fraction~~, the parameter of neurite orientation dispersion and density imaging, can be used as a sensitive~~ had the highest contribution to the diagnosis, thus representing a useful biomarker to detect microstructural changes in the white matter related to neurocognitive and psychiatric disorders in Parkinson’s disease.

Page 6, line 17: “…packing density of axons in WM and spatial organization of the axons, respectively, which are two separate disentangled facets of FA…”: Separate and disentangled serve as redundant terms to each other. Remove one of these terms to improve clarity.

**Response:** We thank the reviewer for their suggestion. Accordingly, we have deleted “separate” from the sentence, as follows:

**1. Introduction** (Page 5, Line 8-12)

The intracellular volume fraction (ICVF) and the orientation dispersion index (ODI) are indices of NODDI that are assumed to reflect the packing density of axons in WM and spatial organization of the axons, respectively, which are two ~~separate~~ disentangled facets of FA (H. Zhang et al., 2012).

Page 7, section 2.2:
-       Line 38: “…64 isotropic diffusion gradients in the anterior-posterior phase-encoding direction…”: Is this worded correctly? All diffusion gradients chosen for each diffusion-weighted volume in the image were oriented in the same direction? Also, were 64 directions collected for b = 1000 and b = 2000 each, or 32 directions each resulting in 64 total?

**Response:** In this study, DWI was performed by spin-echo echo-planar imaging in the anterior-to-posterior phase-encoding direction and 64 isotropic diffusion gradients were used for each shell (b values = 1000 and 2000 s/mm^2^). To clarify, we have revised the following sentences:

**2.2 MRI acquisition** (Page 6, Line 23-29)

Diffusion-weighted imaging (DWI) was performed by spin-echo echo-planar imaging in the anterior-to-posterior phase-encoding direction. Multishell DWI was performed using two b values (1000 and 2000 s/mm^2^) ~~acquired~~ along 64 isotropic diffusion gradients for each shell. ~~in the anterior–posterior phase-encoding direction with the following~~ The sequence parameters were as follows: repetition time (TR), 3300 ms; echo time (TE), 70 ms; field of view (FOV), 229 × 229 mm; matrix size, 130 × 130; resolution 1.8 × 1.8 mm; slice thickness, 1.8 mm; and acquisition time, 7.29 min.

-       Line 43: DWI is used as an acronym here inappropriately, as it is defined as Diffusion Weighted Imaging above in line 34.

**Response:** Thank you for the comment. We have revised the text as follows:

**2.2 MRI acquisition** (Page 6, Line 30-31)

Acquisition of each ~~DWI~~ diffusion-weighted image was completed ~~at b = 0~~ with a gradient-free image ~~with no diffusion gradient~~ (b = 0).

-       Line 43: Does this sentence imply that after 64 diffusion directions, one b0 image is required total? If only one b0 image was collected, authors should address the inability to correct for signal drift throughout their diffusion sequences as a limitation to the accuracy of their diffusion-based measures (see Vos et al., 2016, <https://doi.org/10.1002/mrm.26124>)

**Response:** In this study, we only obtained one b0 image. To clarify this, we have revised the following sentence. Furthermore, we agree with the reviewer in that there is a limitation regarding the use of only one b0 image. Therefore, we have added this as a limitation of the study.

**2.2 MRI acquisition** (Page 6, Line 30-31)

Acquisition of each ~~DWI~~ diffusion-weighted image was completed ~~at b = 0~~ with a gradient-free image ~~with no diffusion gradient~~ (b = 0).

**4. Discussion** (Page 16, Line 19-21)

Finally, in this study, we only obtained one b = 0 image; thus, it is not possible to correct the signal drift, which causes a global signal decrease with subsequently acquired images. This might have affected the estimation of the diffusion parameters (Vos et al., 2017).

Page 8, section 2.3:
-       Line 10: “…each of 64 different axial, sagittal, and coronal directions.”: This sentence did not help to clarify my understanding of the diffusion directions chosen. To this reviewer, axial, sagittal, and coronal refer to three specific planes of anatomical orientation. How are the 64 “directions” oriented within these 3 planes, and how does this reconcile with the previous statement (page 3 line 38) that all 64 directions were isotropic in the anterior-posterior direction?

**Response:** As indicated by the reviewer, the sentence was confusing. We wanted to explain that to exclude the cases with severe artifacts, we visually assessed the original DWI data of all subjects in axial, sagittal, and coronal views. To avoid confusion, we have revised the text as follows:

**2.3 Diffusion MRI pre-processing** (Page 7, Line 10-122)

We then visually assessed all DWI datasets in the ~~each of 64 different~~ axial, sagittal, and coronal ~~directions~~ views.

-       Paragraph 2: Regarding NODDI measure quality checking and interpretation, did the authors review isotropic volume fraction (fiso) images to ensure the anatomical relevance of their ICVF and ODI maps? Essentially, if fiso is high in a region, ICVF and ODI values are arbitrary. See this discussion in the NODDI google group for a starting point on why ICVF and ODI should not be interpreted in absence of fiso interpretation: <https://groups.google.com/forum/#!searchin/noddi/high$20ficvf%7Csort:date/noddi/OhXUUzBpMHI/9YK5C8isykkJ>

**Response:** In this study, we obtained and reviewed the isotropic volume fraction (ISO) maps of each subject. However, we did not include ISO in further analysis. To clarify this, we have added the following description:

**2.3 Diffusion MRI pre-processing** (Page 7, Line 14-16)

Maps of ICVF, ~~and~~ ODI, and isotropic volume fraction (ISO, Supplementary Figure 1) were generated using Accelerated Microstructure Imaging via Convex Optimization (Daducci et al., 2015).

Page 11, line 53: “…no significant differences in AD or ODI values between…”: I thought it would be helpful to add a similar line to the caption of Figure 1, as I flipped back to this section to double check why ODI was not shown once I had viewed Figure 1.

**Response:** According to your suggestion, we have revised the legend of Figure 1 as follows:

**Figure legends** (Page 31, Line 2-16)

**Figure 1.** Comparison of DTI (FA, MD, AD, and RD) and NODDI (ICVF) measures among the healthy control, PD-woNCPs, and PD-wNCPs groups. Using TBSS analysis, significantly lower FA and ICVF (blue–light blue) and significantly higher MD and RD (red–yellow) values were observed in the PD-woNCPs group compared with the healthy controls. Compared with the healthy controls, the PD-wNCPs group had significantly lower FA and ICVF and higher MD, RD, and AD values. Compared with the PD-woNCPs group, the PD-wNCPs group had significantly lower ICVF and higher MD, RD, and AD values. There were no significant differences in ODI values between the groups. The FA skeleton with FA > 0.2 is shown in green. To aid visualization, results are thickened using the fill script implemented in FSL. DTI, diffusion tensor imaging; FA, fractional anisotropy; HC, healthy control; ICVF, intracellular volume fraction; MD, mean diffusivity; NODDI, neurite orientation dispersion and imaging; ODI, orientation dispersion index; PD-woNCPs, Parkinson's disease without neurocognitive–psychiatric symptoms; PD-wNCPs, Parkinson's disease with neurocognitive–psychiatric symptoms; RD, radial diffusivity.

Page 11 & 12, sections 3.2.1, 3.2.2, 3.2.3: I found it odd that several sentences were repeated word for word between sections. I would prefer these sections to be combined into one section to reduce repetition.

**Response:** Thank you for your valuable opinion. Accordingly, we have combined the sections 3.2.1, 3.2.2, and 3.2.3 as follows:

**3.2 TBSS analysis** (Page 10, Line 27—Page 11, Line 28)

~~3.2.1 Healthy controls vs. PD-woNCPs~~

Figure 1 and Table 2 show the results of TBSS analysis of the DTI and NODDI indices. Significantly (*p*FWE < 0.05) lower FA and ICVF values and higher MD, RD, and AD values were observed in the PD-wNCPs group than in the healthy controls, and lower ICVF values and higher MD, RD, and AD values were observed in the PD-wNCPs group than in the PD-woNCPs group. Changes in all indices were observed across the broad areas of the WM compared with the healthy controls. Changes in the ICVF and MD values predominantly occurred in the posterior portion of the WM, whereas changes in the RD and AD values were observed in the circumscribed areas of the WM compared with the PD-woNCPs group. Compared with the healthy controls, the PD-woNCPs group had significantly (*p*FWE < 0.05) lower FA and ICVF values and higher MD and RD ~~(~~*~~p~~*~~FWE < 0.05)~~ values. Changes in the FA and RD values were observed across the broad areas of the WM, whereas changes in the ICVF and MD values predominantly occurred in the anterior portion of the WM. Details regarding the anatomical regions, peak *t*-values, and peak MNI coordinates of significant clusters are shown in Table 2. There were no significant differences in FA values between the PD-woNCPs and PD-wNCPs groups, in AD values between the PD-woNCPs group and healthy controls, and in ODI values between the PD-woNCPs and healthy controls and between PD-woNCPs and PD-wNCPs.

~~3.2.2 Healthy controls vs. PD-wNCPs~~

~~Figure 1 and Table 2 show the results of TBSS analysis of the DTI and NODDI indices. The PD-wNCPs group had significantly lower FA and ICVF values and higher MD, RD, and AD (~~*~~p~~*~~FWE < 0.05) values, as compared with the healthy controls. Changes in all indices were observed across broad areas of the WM. Details regarding the anatomical regions, peak~~ *~~t~~*~~-values, and peak MNI coordinates of significant clusters are available in Table 2. There were no significant differences in ODI between the PD-wNCPs and healthy control groups.~~

~~3.2.3 PD-woNCPs vs. PD-wNCPs~~

~~Figure 1 and Table 2 show the results of TBSS analysis of the DTI and NODDI indices. PD-wNCPs showed significantly lower ICVF values and higher MD, RD, and AD (~~*~~p~~*~~FWE < 0.05) values, as compared with the PD-woNCPs group. Changes in the ICVF and MD values were observed predominantly in the posterior portion of the WM, whereas changes in the RD and AD values were observed in circumscribed areas of the WM. Details regarding the anatomical regions, peak~~ *~~t~~*~~-values, and peak MNI coordinates of significant clusters are available in Table 2. There were no significant differences in FA and ODI values between the PD-woNCPs and PD-wNCPs groups.~~

Page 13, section 3.5: What did other components from the linked ICA look like? Was ICVF present in multiple components or was it limited to IC1? Inclusion of a figure visualizing the makeup of components output by linked ICA would serve as a useful reference for future studies replicating or extending the work presented here.

**Response:** In the revised manuscript, we have added Supplementary Figure 2 to show the relative weights of modalities in each component of LICA analysis. As demonstrated below, ICVF was present in all components.

**3.5 LICA** (Page 12, Line 12-13)

One component (independent component #1 [IC1]) had significant (*F* = 12.03; corrected *p* < 0.05) main effects on the diagnosis (Supplementary Figure 2).

**Supplementary Figure 2.** Relative weights indicating the contribution of each modality within each component

Page 13, line 42: “…the measure of neurite density had the highest contributions to the main effect of diagnosis.”: Did the authors consider running follow up analysis with ICVF alone to augment their interpretation of ICVF as a “sensitive biomarker to detect microstructural changes in white matter related to neurocognitive and psychiatric disorders in Parkinson’s disease.” ?

**Response:** We have considered that a longitudinal study is needed to confirm ICVF as a biomarker of the changes in the white matter related to neurocognitive and psychiatric disorders in Parkinson’s disease. However, unfortunately, we do not have longitudinal data at present, and we have mentioned this as a limitation of this study.

Minor comments:

- The authors should consider a visualization of their raw data to give readers a reference on how each measure presents in healthy and Parkinson’s disease patients, and to aid in the reader’s assessment of overall data quality.

**Response:** Thank you for the suggestion. Accordingly, we have added the figures of DTI (FA, MD, AD, and RD) and NODDI (ICVF, ODI, and ISO) maps as Supplementary Figure 1. However, it is difficult to differentiate a patient with PD from a healthy control by only visualizing the raw image.

**2.3 Diffusion MRI pre-processing**

(Page 7, Line 14-16)

Maps of ICVF, ~~and~~ ODI, and isotropic volume fraction (ISO, Supplementary Figure 1) were generated using Accelerated Microstructure Imaging via Convex Optimization (Daducci et al., 2015).

(Page 16, Line 18-22)

FA, MD, axial diffusivity (AD), and radial diffusivity (RD) maps (Supplementary Figure 1) were then generated for all subjects using the DTIFIT tool of the FMRIB Software Library 5.0.9 (FSL, Oxford Center for Functional MRI of the Brain, UK; www.fmrib.ox.ac.uk/fsl) to fit the tensor model to each voxel of the DWI data (Basser, Mattiello, & LeBihan, 1994).

**Supplementary Figure 1.** DTI (FA, MD, AD, and RD) and NODDI (ICVF, ODI, and ISO) maps of one healthy control and one patient with Parkinson’s disease. AD, axial diffusivity; FA, fractional anisotropy; ICVF, intracellular volume fraction; MD, mean diffusivity; ISO, isotropic volume fraction; ODI, orientation, dispersion index; RD, radial diffusivity.

**Reviewer: 2**

Comments to the Author
The authors have investigated microstructural white matter alterations measured by diffusion weighted imaging and their association to neurocognitive symptoms in PD, compared to PD without neurocognitive symptoms and healthy controls. The paper is well-written, and the authors describe white matter changes linked to cognitive abnormalities, where intra-cellular volume fraction seems to show the highest contribution to the found abnormalities. The writing is concise and overall relevant, but I do have some concerns.

-In the abstract, you state that you included 20 and 19 PD patients with and without NCPs and 20 healthy controls. However, in the methods and in Table 1, you write 25 healthy controls. Which is correct?

**Response:** Thank you for highlighting this error. In this study, we included 25 healthy controls. We have corrected the Abstract.

**Abstract** (Page 3, Line 6-9)

The indices of neurite orientation dispersion and density imaging were compared between 20 and 19 patients with Parkinson’s disease with and without neurocognitive and psychiatric disorders, respectively, and ~~20~~ 25 healthy controls using tract-based spatial statistics and tract-of-interest analyses.

-In multiple places, you state that your results suggest that ICV can be used as a sensitive biomarker to detect microstructural changes. Have you tested this sensitivity in detection of microstructural changes in this paper? If you have not, I suggest that you change this wording. You also say in the discussion (page 16), that “…a study of patients in the prodromal stage of PD is required to determine whether NODDI is sufficiently sensitive to reflect the pathology in the WM related to the NCPs of PD patients”, which contradicts your above statement.

**Response:** In this study, we did not test the sensitivity of each parameter in the detection of microstructural changes in the white matter because the sample size was too small to perform ROC analysis according to Obuchowski, AJR 2000. However, we agree that the term “sensitive” is not appropriate in this context. Thus, we have revised the text as follows:

**Abstract** (Page 1, Line 21-23)

Linked independent component analysis suggested that the intracellular volume fraction can be used as a ~~sensitive~~ useful biomarker to detect microstructural changes in the white matter related to neurocognitive and psychiatric disorders in Parkinson’s disease.

**4. Discussion** (Page 15, Line 9-11)

Together, these results suggest that ICVF is a ~~sensitive~~ useful measure of group differences and a biomarker for the detection of microstructural changes in the WM related to the NCPs of patients with PD.

**5. Conclusions** (Page 16, Line 28-30)

Furthermore, the LICA results showed that ICVF can be used as a ~~sensitive~~ useful measure for the evaluation of microstructural changes in the WM related to the NCPs of patients with PD.

-On page 6, lines 10-13, you summarize studies demonstrating superiority of NODDI over DTI. In this summary, you mention a study by Surova et al, which actually uses another variant of neurite density imaging. So, despite being relatively similar to NODDI, it is not really true that it demonstrates NODDI superiority over DTI. It is however a relevant study to mention in your paper, but maybe with some rephrasing.

**Response:** Thank you for your suggestion. We have revised the text as follows:

**1. Introduction** (Page 5, Line 12-17)

Previous studies have demonstrated the superiority of NODDI over DTI for the detection of PD pathology in the substantia nigra (Kamagata et al., 2016), nigrostriatal pathway (Andica et al., 2018), ~~as well as the WM (Surova et al., 2016)~~ and gray matter (GM) (Kamagata et al., 2017). A simplified model of NODDI, neurite density imaging, has also been used to demonstrate reduction in the density of some WM areas of patients with PD (Surova et al., 2016).

-In table 1 you report H&Y score as mean ± SD. This is inappropriate since H&Y is an ordinal variable with few levels. Please change to reporting frequencies (N/N/N/N/N). The statistic test should for testing differences in H&Y should then also be changed to chi-square.

**Response:** Thank you for the suggestion. Accordingly, we have revised the Table 1 (Page 25) as follows:

**Table 1.** Demographic characteristics of the participants

|  | **HC** | **PD-woNCPs** | **PD-wNCPs** | ***P*-value** |
| --- | --- | --- | --- | --- |
|  |  |  |  |  |
| Number | 25 | 19 | 20 | N/A |
| Age, mean ± SD (years)* | 67.88 ± 2.11 | 67.21 ± 8.16 | 70.15 ± 4.03 | 0.18 |
| Sex (male/female)^†^ | 10/15 | 6/13 | 12/8 | 0.18 |
| Disease duration, mean ± SD (years)^‡^ | N/A | 9.84 ± 6.21 | 9.95 ± 7.69 | 0.79 |
| Hoehn and Yahr scale (1/2/3/4/5), number^~~‡~~†^ | N/A | ~~2.15 ± 0.76~~ 3/11/4/1/0 | ~~2.45 ± 0.83~~ 2/9/7/2/0 | ~~0.24~~ 0.68 |
| MDS-UPDRS part I.1-I.6, mean ± SD | N/A | 0 | 2.90 ± 2.49 | 0.000000012 |
| MDS-UPDRS part III^‡^ | N/A | 17.42 ± 12.28 | 18.60 ± 9.99 | 0.55 |
| Total LED° | N/A | 908.84 ± 444.54 | 817.85 ± 469.99 | 0.54 |
| Total white matter volume, mean ± SD (mL)* | 449.02 ± 53.98 | 452.92 ± 44.88 | 468.58 ± 50.11 | 0.57 |

HCs, healthy controls; LED, levodopa equivalent dose; N/A, not applicable; PD-woNCPs, Parkinson's disease without neurocognitive and psychiatric symptoms; PD-wNCPs, Parkinson’s disease patients with neurocognitive and psychiatric symptoms; MDS-UPDRS, the Movement Disorder Society-Unified Parkinson’s Disease Rating Scale.

*Notes*: Statistical analyses were performed using one-way ANOVA test*, χ^2^ test^†^, Mann–Whitney test^‡^, and unpaired t-test°.

-In “2.2 MRI acquisition” you also say “Acquisition of each DWI was completed at b = 0 with no diffusion gradient.” This is unclear and should be rephrased. How many b = 0 volumes were acquired, and how were they distributed? Were they interspersed?

**Response:** In this study, we only acquired a gradient-free image (b = 0). To clarify this, we have added the following description:

**2.2 MRI acquisition** (Page, Line)

Acquisition of each ~~DWI~~ diffusion-weighted image was completed ~~at b = 0~~ with a gradient-free image ~~with no diffusion gradient~~ (b = 0).

In “2.4 TBSS analysis” you state that “First, FA images of all subjects were affine-transformed into 1 x 1 x 1 mm3…” “…using the FMRIB’s nonlinear registration tool…”. Affine means linear, and cannot be done with FNIRT, so some correction is needed here.

**Response:** Thank you for the correction. We have revised the sentence as follows:

**2.4 TBSS analysis** (Page 7, Line 32—Page 8, Line 2)

First, we performed nonlinear registration of FA images of all subjects ~~affine-transformed~~ into 1 × 1 × 1 × mm^3^ Montreal Neurological Institute (MNI 152) common space (a normalized/averaged brain) using the FMRIB's nonlinear registration tool (Jenkinson et al., 2012).

In page 9, line 3, you state “Without initial registration, the maps were projected onto the mean FA skeleton”. Do you really project maps from individual subjects onto a mean FA skeleton without any prior registration? This is unclear and needs to be better explained.

**Response:** As indicated by the reviewer, this sentence may be misleading. Therefore, we have revised the text as follows:

**2.4 TBSS analysis** (Page 8, Line 6-9)

Finally, ~~a similar process was conducted for~~ the NODDI (ICVF and OD) and DTI (MD, AD, and RD) maps~~. Without initial registration, the maps~~ were projected onto the mean FA skeleton after applying the warping registration field of each subject to the standard space.

In “2.8 Statistical analysis”, the TBSS section only mentions ANOVA techniques, but from your results, it seems that pairwise comparisons were also conducted from the TBSS (sections 3.2.2 and 3.2.3). This needs to be clearer.

**Response:** Thank you for your comment. To clarify this, we have revised the text as follows:

**2.8 Statistical Analysis** (Page 9, Line 21-24)

For TBSS analyses, a voxel-wise GLM framework with one-way ANOVA followed by pairwise post hoc comparisons, which included age and sex as covariates, was applied to compare all diffusion metrics among groups (healthy controls vs. PD-woNCPs vs. PD-wNCPs) using the FSL randomize tool with 5000 permutations.
